# The complete mitochondrial genome sequence and phylogenetic position of *Gobio coriparoides*

**DOI:** 10.1080/23802359.2020.1715864

**Published:** 2020-01-24

**Authors:** Yushuang Ge, Qiqun Cheng, Yongbin Yan, Xiaochen Duan, Tai Wang, Yanping Zhang, Zhongyu Lou, Yanyan Du

**Affiliations:** a College of Marine Sciences, Shanghai Ocean University, Shanghai, China;; b Key Laboratory of Oceanic and Polar Fisheries, Ministry of Agriculture and Rural Affairs, East China Sea Fisheries Research Institute, Chinese Academy of Fishery Sciences, Shanghai, China;; c Gansu Fisheries Research Institute, Lanzhou, China

**Keywords:** *Gobio coriparoides*, mitochondrial genome, phylogenetic analysis, molecular evolution, genetic structure

## Abstract

In this paper, we determined the complete mitochondrial DNA (mtDNA) sequence of *Gobio coriparoides* and analyzed its phylogenetic position. The complete mitogenome is 16,604 bp in length. It consists of 13 protein-coding genes, 2 rRNA genes, 22 tRNA genes, and 1 control region. Among the 37 genes, 28 were encoded on the heavy strand, while 9 were encoded on the light strand. The overall base composition was 28.97% for A, 18.06% for G, 26.54% for T, and 26.43% for C, with a higher A + T content (55.51%). There are some overlaps existing in *G. coriparoides* mitochondrial genome. The neighbor-joining (NJ) phylogenetic tree based on whole mitogenome sequences supported that *G. coriparoides* is the closest to *G. cynocephalus*. This result will provide a basic reference for understanding the genetic structure, molecular evolution, and phylogeny of *G. coriparoides* and related species.

*Gobio coriparoides*, which belongs to the subfamily Gobioninae, family Cyprinidae, and order Cypriniformes, is mostly distributed in the second largest river of China, i.e. Yellow River (Wang 1991). *Gobio coriparoides* is very sensitive to circumstance around its habitat and accordingly become an environmental indicator. In the recent decades, its population has decreased noticeably due to environmental degradation. Many basic data including genetic information are urgently needed for the protection of *G. coriparoides*. The complete mitogenome sequence information of *G. coriparoides* can provide useful data for further studies on genetic structure, molecular evolution, and phylogeny of *G. coriparoides* and related species.

In this study, *G. coriparoides* was collected from Yanguo Gorge (36°3′6″N, 103°15′29″E), the upper of Yellow River, and was deposited in herbarium at East China Sea Fisheries Research Institute with voucher number ECSFRI-STJ01. We obtained the complete mitochondrial genome sequence of *G. coriparoides* using the primer-walking method and deposited it into the GenBank database with an accession number MN864250.

The whole mitogenome length of *G. coriparoides* was determined to be 16,604 bp. It contains13 protein-coding genes (ATP6 and 8, COI-III, Cytb, ND1-6 and 4 L), 22 transfer RNA genes (tRNAs), 2 ribosomal RNA genes (12S and 16S rRNAs), and 1 control region. Except for one protein-coding gene ND6 and eight tRNAs (tRNA^Gln^, tRNA^Ala^, tRNA^Asn^, tRNA^Cys^, tRNA^Tyr^, tRNA^Ser^, tRNA^Glu^, and tRNA^Pro^), all other genes are encoded on the heavy strand, which are in accordance with the other teleost mitogenomes (Miya et al. 2003). The overall nucleotide base composition of *G. coriparoides* mitogenome is as follows: A, 28.97%; G, 18.06%; T, 26.54%, and C, 26.43%, with A + T content 55.51%, showing an obvious anti-G bias as appeared in other vertebrates (Qiao et al. 2013). All protein-coding genes in *G. coriparoides* use ATG as the start codon except for COI, which starts with GTG. There are seven genes (COI, ATP8, ATP6, ND3, ND4L, ND5 and ND6) ending with TAA whereas two (ND1 and ND2) with TAG. The remaining four genes have an incomplete stop codon: T (COII and Cytb) and TA (COIII and ND4), respectively.

There are some overlaps existing in *G. coriparoides* mitochondrial genome. Between the 13 protein-coding genes of *G. coriparoides* mitochondrial genome, four overlaps were detected as shown in ATP8-ATP6, ATP6-COIII, ND4L-ND4, and ND5-ND6. Among 22 tRNAs, two overlaps are found in tRNA^Ile^-tRNA^Gln^ and tRNA^Thr^-tRNA^Pro^. Furthermore, the pairs of genes ND2-RNA^Trp^, COIII-tRNA^Gly^, and ND3-tRNA^Arg^ also revealed overlaps.

To further investigate its phylogenetic position, 11 complete mitochondrion genomes of Cyprinidae were downloaded from NCBI GenBank. A neighbor-joining (NJ) tree was constructed using Mega 7.0 (Kumar et al. 2016). It shows that *G. coriparoides* is closest to *G. cynocephalu*s, then together with *G. gobio* forming a monophyletic group (Figure 1).

**Figure 1.**
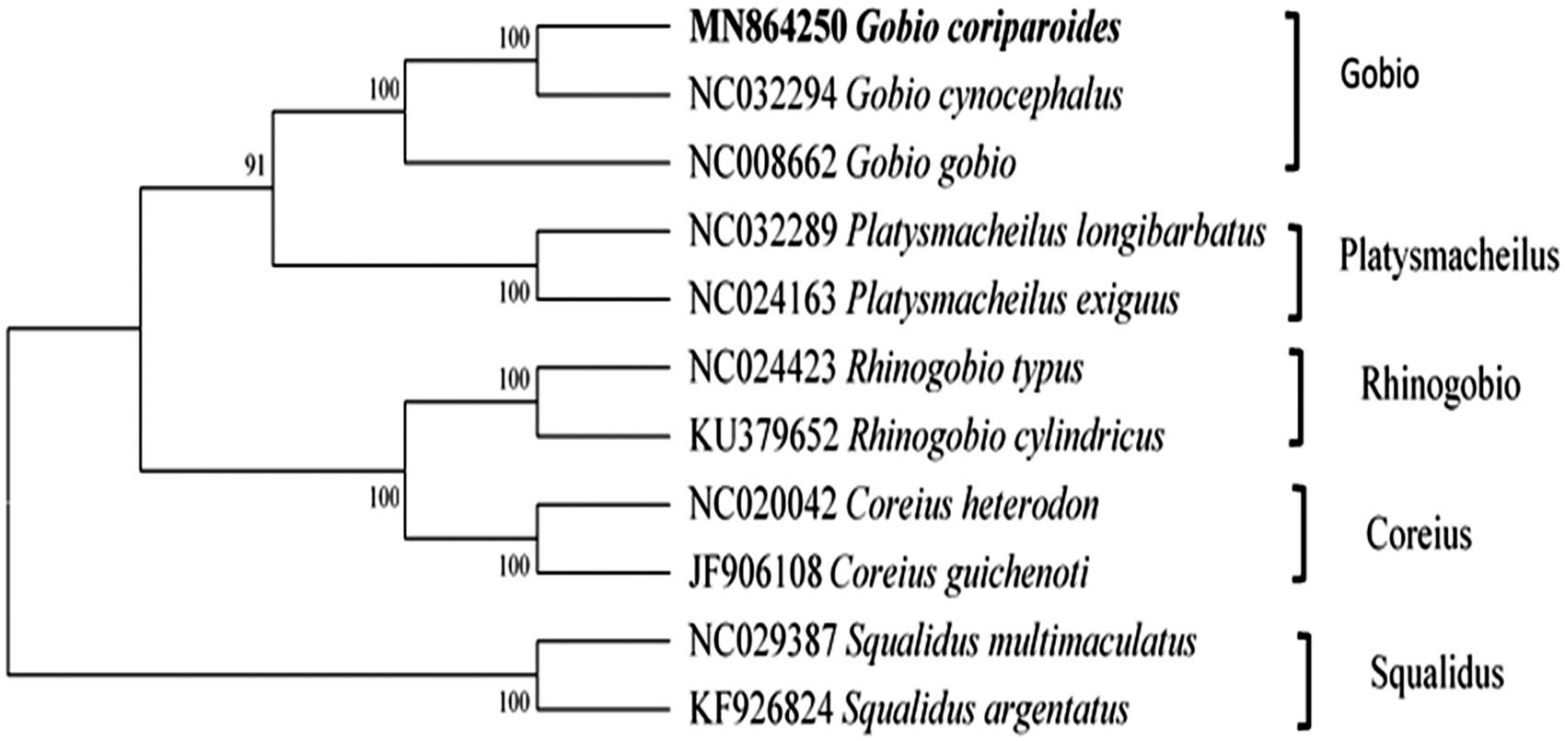
Phylogenetic relationship of 11 species within Cyprinidae based on whole mitogenome sequences using the NJ method. *G. coriparoides* is highlighted with bold font.
